# Application of fluorescence correlation spectroscopy to study substrate binding in styrene maleic acid lipid copolymer encapsulated ABCG2

**DOI:** 10.1016/j.bbamem.2020.183218

**Published:** 2020-06-01

**Authors:** Aaron J. Horsey, Deborah A. Briggs, Nicholas D. Holliday, Stephen J. Briddon, Ian D. Kerr

**Affiliations:** a School of Life Sciences, University of Nottingham, Queen's Medical Centre, Nottingham NG7 2UH, UK; b Centre of Membrane Proteins and Receptors, University of Birmingham and University of Nottingham, The Midlands, UK

**Keywords:** ABC transporter, Pharmacology, Multidrug resistance, Membrane protein, SMALP, Fluorescence, Fluorescence correlation spectroscopy, Photon counting histogram

## Abstract

ABCG2 is one of a trio of human ATP binding cassette transporters that have the ability to bind and transport a diverse array of chemical substrates out of cells. This so-called “multidrug” transport has numerous physiological consequences including effects on how drugs are absorbed into and eliminated from the body. Understanding how ABCG2 is able to interact with multiple drug substrates remains an important goal in transporter biology. Most drugs are believed to interact with ABCG2 through the hydrophobic lipid bilayer and experimental systems for ABCG2 study need to incorporate this. We have exploited styrene maleic acid to solubilise ABCG2 from HEK293T cells overexpressing the transporter, and confirmed by dynamic light scattering and fluorescence correlation spectroscopy (FCS) that this results in the extraction of SMA lipid copolymer (SMALP) particles that are uniform in size and contain a dimer of ABCG2, which is the predominant physiological state. FCS was further employed to measure the diffusion of a fluorescent ABCG2 substrate (BODIPY-prazosin) in the presence and absence of SMALP particles of purified ABCG2. Autocorrelation analysis of FCS traces enabled the mathematical separation of free BODIPY-prazosin from drug bound to ABCG2 and allowed us to show that combining SMALP extraction with FCS can be used to study specific drug: transporter interactions.

## Highlights

• ABCG2 is solubilised from HEK293T cells into styrene maleic acid lipid copolymers.
• Fluorescence correlation spectroscopy confirms that SMALPs contain an ABCG2 dimer.
• Purified SMALP-ABCG2 binds the known ABCG2 substrate BODIPY-prazosin.
• SMALP-ABCG2 is amenable to pharmacological measurements of ABCG2 in lipid bilayer patches.

## Graphical abstract

**Figure:**
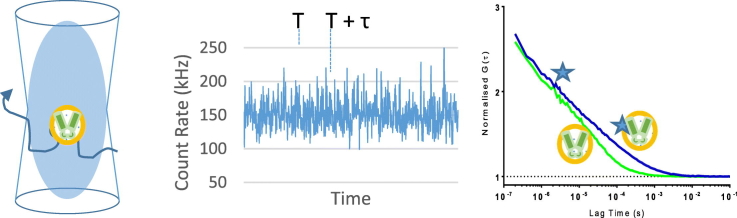

## 1 Introduction

The ATP binding cassette (ABC) family of membrane transporter proteins couple the hydrolysis of ATP at intracellular nucleotide binding domains (NBDs) to the binding and transport of substrates across the membrane. They have a phenomenally diverse array of physiological roles including nutrient uptake in bacteria, hormone transport in plants, and bile salt and antigenic peptide transport in animals [1]. Several family members are capable of exporting a wide range of chemically diverse compounds from the cell. This unusual polyspecificity underpins roles in cell, tissue and organ level defence [2], but in disease states these polyspecific transporters can underlie the emergence of a treatment refractory state. Such multidrug resistance (MDR) to chemotherapy drugs can be a contributory factor to poor prognosis in cancer [3,4]. Three human MDR-type ABC transporters (P-glycoprotein (ABCB1), multidrug resistance associated protein-1 (ABCC1/MRP1) and breast cancer resistance protein (ABCG2/BCRP)) have been the subject of intensive investigation both to understand their contribution to cancer MDR and to understand the protein biochemical mechanisms of multidrug recognition and export [[5], [6], [7]].

ABCG2 has specifically been implicated in conferring a cytoprotective role in many types of stem cells under conditions of cellular stress (e.g. hypoxia [8,9]), and it also appears to be involved in the cellular stress response in autophagy [10]. ABCG2 overexpression has been linked to poor prognosis in several different haematological malignancies [[11], [12], [13]], and altered function of ABCG2 due to inherited polymorphisms is a major risk factor for hyperuricaemia [[14], [15], [16]].

This plethora of physiological roles indicates that ABCG2's substrate repertoire is diverse. To date, using transport assay screens [17,18], ABCG2 has been demonstrated to be capable of transporting camptothecins, polyglutamates, statins, anthracyclines and nucleoside analogues amongst others. A similarly wide range of small molecules appear capable of inhibiting ABCG2 such as tyrosine kinase inhibitors, immunosuppressants, HIV protease inhibitors and calcium channel blockers [14,19]. These lists, which include scores of pharmaceutically useful drugs, implicate ABCG2 as a major contributor to drug uptake and elimination. Understanding the molecular basis of ABCG2's complex pharmacology is therefore paramount. Early studies demonstrated that ABCG2 has multiple, pharmacologically distinct sites that are allosterically linked to each other, and to the NBDs [20,21]. Recent cryo-electron microscopy structural data have led to the identification of cavities within the transporter at which substrates and inhibitors can interact [[22], [23], [24]] providing a framework for understanding structure activity relationships for existing and novel ABCG2 substrates and inhibitors [[25], [26], [27]].

Quantitative determination of the binding of substrates and inhibitors to ABCG2 will complement structural, theoretical and medicinal chemical approaches to better describe ABCG2 and will result in a molecular understanding of its roles in physiology and pathology. As the majority of ABCG2 transport substrates are hydrophobic and are expected to interact via the lipid milieu it is essential that any system for determining pharmacology includes surrounding lipids. This limits studies using detergent solubilised protein as this would remove all but the most tightly associated lipids. Styrene maleic acid (SMA) has emerged as an adjunct to existing methods of membrane protein extraction [28,29]. It has been demonstrated to extract a huge array of target membrane proteins from both prokaryotes and eukaryotes into a near native lipid environment with a lipid shell whose composition reflects that of the original membranes [28,30].

Herein, we describe a solution-based method using fluorescence correlation spectroscopy (FCS) to determine the binding of substrate to ABCG2 in a native-like lipid environment. FCS is an optical technique which analyses the intensity fluctuations generated as a fluorescent species diffuses though a small, defined confocal volume (~0.2 fL) to generate information about its concentration, diffusion and brightness [31,32]. Following styrene maleic acid co-polymer extraction and purification of ACBG2, we were able to use FCS and photon counting histogram (PCH) analysis to confirm that the protein retains a predominantly homodimeric structure in membrane patches. The solubilised and purified protein in SMALP co-polymers is amenable to quantitative studies of drug interaction using FCS, because concentrations of fluorescent drug molecules in solution and fluorescent drug bound to ABCG2 can be simultaneously quantified by virtue of their different diffusion coefficients. The combination of SMALP purification and FCS analysis will enable future solution-based pharmacology of ABCG2 and other membrane proteins.

## 2 Methods

### 2.1 Molecular biology

To enable protein purification, existing pcDNA3.1(+)zeo vectors (Invitrogen) encoding N-terminally superfolder GFP-tagged ABCG2, CD28, CD86 [33] and SNAP-tagged ABCG2 (NCBI Gene 9429) were modified by the insertion of a start codon and hexahistidine tag, 5′ to the original start codon of the GFP/SNAP tag. Polyhistidine tagging was accomplished by annealing phosphorylated single-stranded oligonucleotides with 5′ (CTAG) and 3′ (GATC) extensions that enabled insertion at an existing *Nhe*I restriction site. The original methionine start codon of the GFP/SNAP tags was subsequently modified by primer based Quikchange site directed mutagenesis to a leucine, and all constructs were sequenced across the entire reading frame (Source Bioscience, Nottingham, UK).

### 2.2 Cell culture

HEK293T cells (ATCC CRL-3216) were maintained and transfected by polyethyleneimine as previously described [[33], [34], [35]]. Stable cell lines were selected in the presence of 200 μg/mL zeocin (ThermoFisher) and then routinely passaged in the presence of 40 μg/mL zeocin to maintain transgene expression. High expressing cell lines were established by growing clones in multiwell plates, labelling His-SNAP-ABCG2 with 0.3 μM SNAP-Cell TMR-Star (New England Biolabs) and imaging using an ImageXpress Micro XLS high content analysis system (Molecular Devices) equipped with TRITC excitation/emission filter sets and a Zeiss 20× long working distance air objective. The obtained images were manually assessed for relative brightness as an indicator of protein expression level. Protein expression was further enhanced by the addition of sodium butyrate (10 mM) to cell cultures 24 h prior to harvesting. To facilitate greater cell densities and yields for protein expression, stably transfected His-SNAP-ABCG2-expressing HEK293T cells were adapted to suspension culture by agitation at 180 rpm in flat-bottomed borosilicate vessels with high levels of cell viability (>95%) maintained in media (4.5 g.L^−1^ glucose Dulbecco's modified Eagle medium; DMEM) supplemented with 10% v/v foetal bovine serum (FBS). Final densities of ~2 × 10^6^ cells/mL were routinely achieved with suspension cell culture.

### 2.3 Mitoxantrone accumulation assay

The function of N-terminally SNAP-tagged ABCG2 was verified by a previously described assay in which expression of the transporter limits intracellular accumulation of the fluorescent substrate mitoxantrone [33]. Briefly, confluent monolayers of HEK293T cells in 96-well plates (655090, Greiner Bio-One, Stonehouse, UK) were incubated with vehicle (0.1% v/v DMSO), 8 μM mitoxantrone (MX; Sigma-Aldrich, Poole, UK), or mitoxantrone plus the ABCG2 inhibitor Ko143 (1 μM; Sigma-Aldrich) for 2 h at 37 °C 5% CO_2_. Cells were washed twice with ice-cold PBS, fixed with 4% w/v paraformaldehyde, and cellular fluorescence measured using a Flexstation (Molecular Devices) with excitation wavelength 607 nm and emission 684 nm. The percentage Ko143 inhibitable mitoxantrone accumulation was calculated as follows, where I_MX and Ko143_ is the vehicle corrected fluorescence intensity of cells treated with both MX and Ko143, and where I_MX_ is the vehicle corrected fluorescence intensity of cells treated with MX alone.

$$\%=\frac{I_{\mathrm{MX}\;\mathrm{and}\;\mathrm{Ko}143}-I_{\mathrm{MX}}}{I_{\mathrm{MX}}}\times 100$$

### 2.4 SMALP extraction and protein purification

Whole cell membranes were obtained from cells using a 2-step centrifugation protocol. Cell pellets were resuspended at 5–10 pellet volumes in membrane isolation buffer (MIB; 10 mM Tris, 0.25 M sucrose, 0.2 mM CaCl_2_, pH 7.4) supplemented with protease inhibitors (protease inhibitor cocktail III, Calbiochem). Cells were then disrupted by nitrogen cavitation (1000 psi, 15 min, 4 °C, Parr Instrument Company) and cellular debris collected by centrifugation (1500*g*, 15 min, 4 °C). The supernatant was applied to thin-walled polypropylene tubes and ultracentrifuged for 45 min at 100,000*g*, 4 °C. The pelleted whole cell membranes were resuspended in MIB (omitting the CaCl_2_) by repeated shearing through a 25G needle. Cell membranes were snap-frozen at −80 °C in aliquots. SMA2000 (Cray Valley) was prepared as previously described [36]. SMALP solubilisation was performed using membrane pellets centrifuged (100,000*g*, 20 min, 4 °C) and resuspended at 100 mg wet membrane weight/mL SMA buffer (150 mM NaCl, 50 mM Tris, pH 8.0). SMA was added at a final concentration of 2.5% w/v and the suspension was incubated at room temperature (18–22 °C) for 1 h with gentle rotation. The solubilised protein was obtained via ultracentrifugation (100,000*g*, 20 min, 4 °C) and used without further storage.

Solubilised His-tagged protein was mixed with His-select Cobalt resin (Sigma) at a ratio of 100 μL resin per 1 mL of solubilised protein with end-over-end mixing overnight at 4 °C. The mixture was then poured into a gravity flow column (Bio-Rad) and the flow through collected. The resin was washed thrice with 5 bed volumes of SMA buffer and subsequently with SMA buffer supplemented with 50 mM, 200 mM and 2 M imidazole (each additional step comprising three separate washes of 2 bed volumes). Protein containing fractions were identified by SDS-PAGE analysis (using BXP-21 antibody (Merck) at 1:500 dilution or anti-His-HRP conjugate (R&D Systems) at 1:1000 dilution on western blots where necessary). Protein containing fractions were pooled, filtered (0.22 μm) to remove particulate contaminants, and then concentrated in 10 kDa molecular weight cut-off concentrators (Sartorius Vivaspin) to a 10% final volume. Imidazole was removed by back-dilution and concentration in imidazole free SMA buffer. Protein concentration was determined using densitometric analysis of SDS-PAGE gels containing a standard curve of bovine serum albumin alongside fractions of purified ABCG2.

### 2.5 SNAP-tag labelling

In intact cells, SNAP-tag labelling was achieved by incubating cells for 30 min at 37 °C in a 5% CO_2_ atmosphere with 1 μM SNAP-Cell Oregon Green (all SNAP labels were from New England Biolabs) followed by washing thrice with media prior to imaging. Specificity of SNAP-tag labelling was ensured by controls pre-incubated with 2 μM SNAP-Cell-Block under identical conditions. For labelling of purified SNAP-tagged protein, membranes were resuspended at 100 mg wet weight per mL of SMA buffer and incubated with 0.5 μM SNAP-Surface AlexaFluor (AF) 647 for 2 h at room temperature, prior to solubilisation with SMA and purification as described above.

### 2.6 Confocal microscopy

To confirm expression and membrane localisation of protein constructs cells were imaged live using confocal microscopy. Cells were seeded at 2.5 × 10^5^ cells/well in 35 mm glass bottom dishes (MatTek Corp) 24 h prior to imaging. Cells were subsequently washed twice with pre-warmed (37 °C) phenol-red free HBSS (Hank's Balanced Salt Solution, Sigma-Aldrich) immediately prior to imaging on a LSM710 confocal laser scanning microscope (Carl Zeiss, Jena, Germany), using a Plan-Apochromat 63×/1.40 Oil Ph3 DIC M27 objective. Images (1024 × 1024 pixels with 8-bit image depth) were collected using an argon laser, with excitation wavelength of 488 nm and emission collected either at 500-550 nm (GFP-tagged proteins) or 493-598 nm (SNAP-AF488 labelled proteins) using a 90 μm pinhole. Gain and offset settings were adjusted to maintain signal within the linear range of the detector and were maintained within each experiment.

### 2.7 DLS

Hydrodynamic measurements were made using a Zetasizer Nano instrument. SMALPs were diluted 10-fold in SMA buffer, equilibrated to 25 °C, and data then collected for 10s per read with 5–10 reads per sample. Mean particle hydrodynamic radii and relative component proportions were calculated from at least three independent preparations of SMALP solubilised proteins.

### 2.8 Fluorescence correlation spectroscopy (FCS) measurements

FCS measurements were performed on a Zeiss LSM510NLO Confocor 3 (Carl Zeiss, Jena, Germany) using a 40× c-Apochromat 1.2 NA water-immersion objective using either argon (488 nm excitation) or helium‑neon (HeNe) (633 nm excitation) lasers.

The microscope was aligned and calibrated on each experimental day using rhodamine 6G (for 488 nm-excitation beampaths) or Cy5-NHS ester (for 633 nm excitation beampaths) as previously described [33,37,38] to allow calculation of the measurement volume and subsequent sample concentrations and diffusion coefficients. Solutions containing fluorescently tagged protein or compound were added to the wells of a Nunc™ Lab-Tek™ 8-well chambered #1.0 cover glass (ThermoFisher). The samples were prepared in a final volume of 150-300 μL depending on the requirement for the experiment. BODIPY-Prazosin (ThermoFisher) or GFP tagged proteins were excited with 488 nm laser and emission collected with a 505-550 nm bandpass filter. SNAP Surface Alexa Fluor® 647 labelled samples were excited with 633 nm laser and emission was collected through a 650 nm longpass filter. In both cases a pinhole diameter of 1 Airy Unit (70 μm for 488 nm excitation, 90 μm for 633 nm excitation) was used. Fluorescence intensity fluctuations were collected for 3 × 10–100 s as indicated using a laser power of ~0.3 kW/cm^2^.

### 2.9 Data analysis

FCS data were analysed using autocorrelation (AC) or photon counting histogram (PCH) analysis using Zen 2012 software (Carl Zeiss, Jena, Germany). Autocorrelation curves for both direct measurement of SMALP purified ABCG2 and BODIPY-prazosin binding were fitted using a two-component 3D diffusion model incorporating a pre-exponential triplet term to account for fluorophore photophysics, with triplet lifetime constrained to <10 μs. For all fits, the structure parameter was fixed to that measured in the calibration read for the appropriate wavelength. For direct measurements of SMALP-purified ABCG2 dwell times for both components were allowed to vary freely, with component one (τ_D1_) constrained to 300-600 μs representing diffusion of single SMALPs as determined from preliminary experiments. For BODIPY-prazosin binding experiments, dwell time for component 1 (τ_D1_) was fixed to that of free BODIPY-prazosin as measured directly at 20 nM in solution (40-60 μs), with the second component (τ_D2_) constrained to 180-400 μs, as determined from direct measurements of SMALP-purified ABCG2. For multicomponent fits, concentrations of each component were calculated from their fractional contributions towards the total particle number and converted to concentrations using the calibrated volume size as previously described [37]. Photon counting histograms were generated from fluorescence fluctuations in Zen 2012 using a 20 μs bin time, and fitted to a two-component PCH model, with a first order correction fixed to that determined from PCH analysis of the rhodamine calibration data.

## 3 Results and discussion

### 3.1 Solubilisation of ABCG2 into styrene maleic acid lipid co-polymers

Supplementary Fig. 1

### 3.2 ABCG2 is dimeric in SMALPs

Determination of ABCG2 function in SMALPs first requires validation that the protein is in a physiological oligomeric state. Homo-dimerisation of this transporter is the minimal oligomeric state essential for function [19,44] and cryo-EM has provided structural confirmation of the homodimer [[22], [23], [24]]. Fluorescence correlation spectroscopy and photon counting histogram (PCH) analysis, together with stepwise photobleaching experiments have also provided evidence of ABCG2 oligomers in the plasma membrane of living cells [33]. Here, FCS and PCH were used to determine the diffusion characteristics and oligomeric state of SMALP-purified monomeric and dimeric control proteins (CD28 and CD86, respectively) and compare these to ABCG2 in SMALPs.

**Fig. 1:**
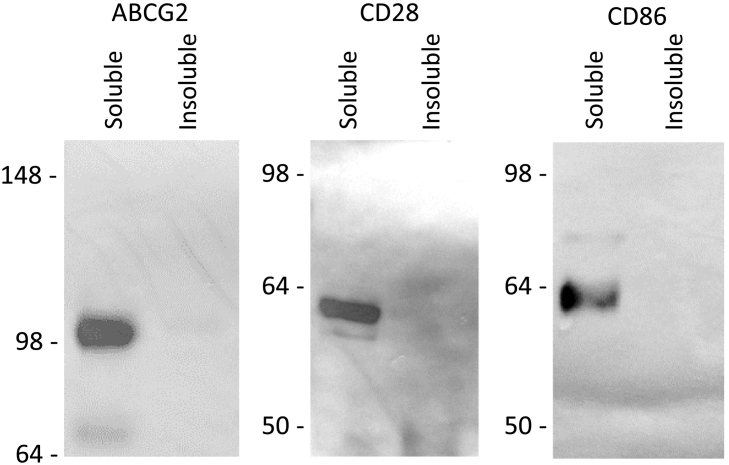
Solubilisation of membrane proteins by styrene maleic acid. GFP-tagged proteins were solubilised from membranes (100 mg wet weight mL^−1^) with 2.5% w/v SMA for 1 h at room temperature prior to ultracentrifugation to separate soluble from insoluble fractions. Insoluble material was resuspended back into the original volume in SMA buffer supplemented with 2% w/v SDS. Blots shown are representative of n = 3 independent experiments.

**Table 1:** Particle size analysis of SMALP encapsulated ABCG2.

Protein	Hydrodynamic radius (nm)	Unit particle frequency (%)
ABCG2	9.8 ± 0.5	96.9 ± 2.5
CD28	9.5 ± 1.0	95.6 ± 3.9
CD86	7.5 ± 1.6	96.5 ± 3.3

Samples of solubilised membrane material from cell lines expressing the given proteins were analysed by DLS. The size distribution showed the predominant particle sizes in each case (n = 3, mean ± SD).

**Fig. 2:**
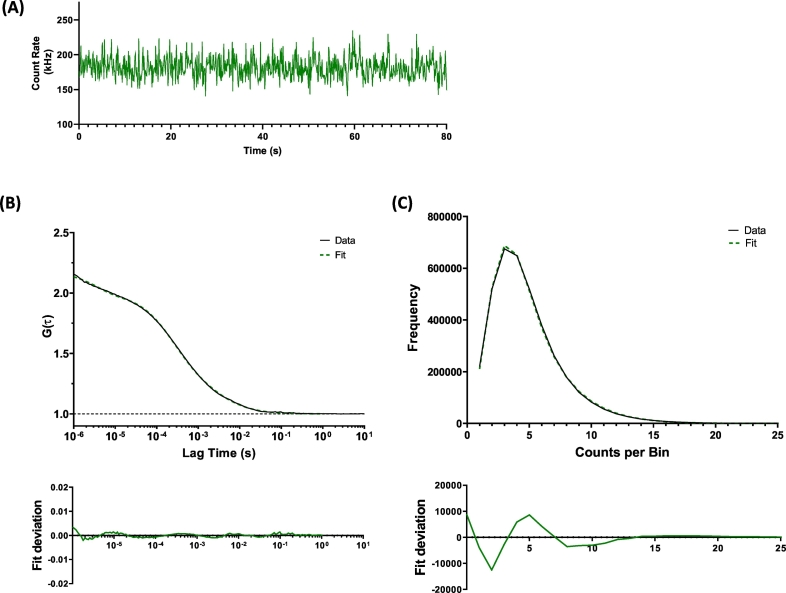
Fluorescence correlation spectroscopy and photon counting histogram analysis of SMALP-purified ABCG2-GFP. SMALPs containing ABCG2-GFP were prepared in assay buffer as described in Methods, and FCS measurements were obtained from 150 μL of sample as described in Methods. (A) Fluorescence fluctuation traces were obtained using 488 nm excitation and analysed by either autocorrelation (AC) analysis or photon counting histogram (PCH) analysis. (B) Autocorrelation curves (*upper*) were best fit by a 2-component 3D diffusion model (residuals shown in *lower*) to obtain an average dwell time and particle number of ABCG2-GFP SMALPs, from which a diffusion coefficient and concentration could be calculated. (C) PCH analysis of the same fluctuations (*upper*) using a 20 μs bin time could be fit (residuals, *lower*) to obtain a value for the molecular brightness of the ABCG2-SMALPs. Data shown is an example read and analysis of a single sample, representative of those described in Table 2.

**Fig. 3:**
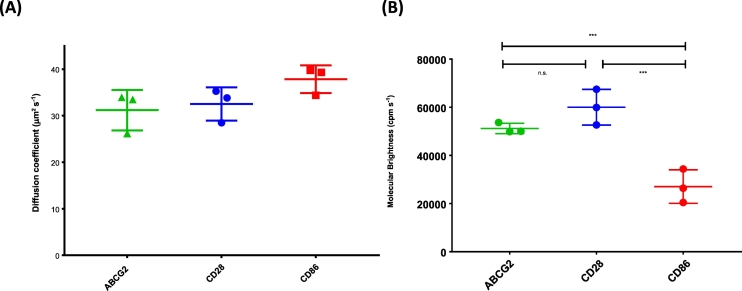
Particle diffusion and brightness analysis by FCS and PCH analysis. (A) The diffusion coefficients of SMALP encapsulated ACBG2 and control proteins CD28 and CD86 were determined by 2-component fitting of the autocorrelation curve (Fig. 2B). The diffusion coefficients of the predominant component (C1; Table 2) in each case were not statistically different to each other. (B) The molecular brightness of the predominant component in PCH analysis of ABCG2 was equivalent to that of the dimeric control (CD28) and approximately double that of the monomer control (CD86). Data represent the mean ± SD of three independent experiments each consisting of 3 measurements and significance is shown following one way ANOVA and Sidak post-analysis.

Fluorescence fluctuation traces for SMALP-encapsulated ABCG2 were initially obtained (Fig. 2A) and autocorrelation analysis performed (Fig. 2B). The autocorrelation curve was best fit using a 2-component curve as indicated by analysis of the fitting residual (Fig. 2B, lower panel). The first of these components had a dwell time (τ_D1_) of approximately 180 μs, which corresponded to a diffusion coefficient (D) of 31.2 ± 4.3 μm^2^s^−1^ (Fig. 3A, Table 2). An additional, slower component with D of 1.8 ± 0.5 μm^2^s^−1^ was also detected in these experiments, but this represented only ~11% of the total sample. Consistent with the DLS data above, this most likely represents aggregated SMALPs. Using this diffusion coefficient in the Stokes-Einstein equation, with the proviso that SMALP particles are not spherical, gives an estimate of hydrodynamic radius of 7–9 nm, consistent with the DLS data. Similar diffusion coefficients for unit SMALP encapsulated CD86 and CD28 were obtained, and confirm the relatively uniform size of extraction by SMA polymer (Fig. 3A; Table 2).

**Table 2:** SMALP diffusion coefficient as calculated by fluorescence correlation spectroscopy and subsequent autocorrelation analysis.

Protein	Component 1		Component 2	
	Fraction (%)	D (μm^2^s^−1^)	Fraction (%)	D (μm^2^s^−1^)
ABCG2	88.5 ± 5.2	31.2 ± 4.3	11.5 ± 5.2	1.8 ± 0.5
CD28	95.7 ± 2.7	32.5 ± 3.6	4.3 ± 2.7	2.8 ± 0.9
CD86	98.7 ± 1.4	37.8 ± 3.0	1.3 ± 1.4	2.1 ± 0.9

FCS data were analysed with a 2-component model as described in the methods to determine diffusion components of SMALP particles in solution. Values given represent the mean ± SD for three independent experiments, with multiple fluctuation reads within each sample.

PCH analysis of ABCG2 fluctuations was also best fit with two components (Fig. 2C), with a less bright component constituting 94.6% of the total particles, with 5.4% of the particles exhibiting a 7.8-fold higher molecular brightness (Table 3). This is consistent with the autocorrelation and DLS data and suggests that the brighter component represents aggregated SMALPs and the majority component representing individual SMALP particles. Photon counting histogram (PCH) analysis of monomeric (CD86) and dimeric (CD28) standards were also compared to investigate the stoichiometry of ABCG2 protein in the SMALP particles (Fig. 3B). As with ABCG2, both samples showed a predominant (>90%) bright component with small amount (<7%) of 8–10-fold brighter species.

**Table 3:** PCH analysis of SMALP encapsulated ABCG2, and monomeric (CD86) and dimeric (CD28) controls.

Protein	Component 1		Component 2	
	Fraction (%)	ε (cpm.s^−1^)	Fraction (%)	ε (cpm.s^−1^)
ABCG2	94.6 ± 1.6	51,999 ± 2135	5.4 ± 1.6	408,163 ± 12,498
CD28	93.8 ± 1.3	60,030 ± 7406	6.2 ± 1.3	465,789 ± 71,770
CD86	93.0 ± 1.5	27,074 ± 6975	7.0 ± 1.5	271,957 ± 47,354

Fluorescence fluctuations were analysed by PCH analysis using a 2-component model as described in the text. Values given represent the mean ± SD for three independent experiments.

Analysis of component 1 of the PCH analysis for all three SMALP species (Fig. 3B) indicated that the molecular brightness (ε, counts per molecule per second) of CD86 (monomeric control protein [33,40]) was 27,074 ± 6975 cpms^−1^ (n = 3) whilst that for CD28 (dimeric control) was 2.2-fold higher (60,030 ± 7406 cpms^−1^, n = 3).This confirmed that PCH analysis of SMALP encapsulated proteins could correctly determine the relative stoichiometry of monomeric and dimeric states (Table 3). In comparison to CD28 and CD86, the molecular brightness for the equivalent component of the ABCG2 PCH analysis was 51,199 ± 2135 cpms^−1^ (n = 3), which was 1.9-fold that of the monomeric CD86, and not significantly different from that of CD28 (p = 0.25), demonstrating that ABCG2 is essentially dimeric within the SMALP particle (Fig. 3B). The consistency of SMALP particle sizes excludes the possibility that variations in overall SMALP size and/or heterogeneity could contribute to differences seen in molecular brightness measurements. Confirmation of ABCG2's (and CD28's) dimeric status in SMALPs adds to accumulating evidence that SMA extracts membrane proteins of diverse folds and functions into particles containing the native oligomer. For example, AcrB was extracted as a trimer [45] and KcsA has been extracted as a tetramer [46].

### 3.3 N-terminal tagging of ABCG2 with a SNAP-tag does not impair localisation or function

**Fig. 4:**
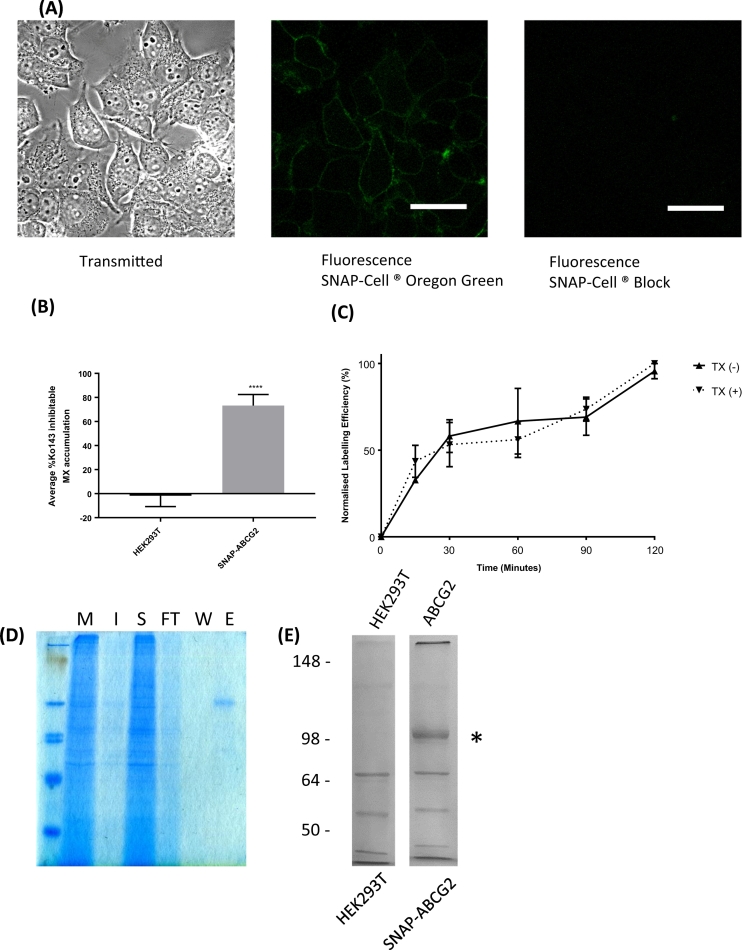
SNAP-ABCG2 surface expression, activity and purification. SNAP-ABCG2 expressing cells were labelled for 30 min at 37 °C 5% CO2 with 1 μM SNAP-Cell Oregon Green alone (middle) or after pre-incubation with 2 μM SNAP-Cell ® Block (right panel). After washing cells were imaged using an LSM710 confocal microscope (Carl Zeiss) with fluorescence images gathered using 488 nm/493-598 nm excitation/emission wavelengths. Scale bar = 20 μm. (B) Corrected mitoxantrone (MX) fluorescence intensity values were compared in the presence and absence of Ko143 and the function of ABCG2 was determined as % Ko143 inhibitable MX accumulation. Data are plotted as mean ± SD (n = 4) with statistical significance (****, p < 0.001) compared to parental HEK293T assessed by unpaired *t*-test. (C) Labelling of SNAP-ABCG2 in membrane fractions prior to purification. Membranes were incubated with SNAP-Surface AlexaFluor® 647 in the presence (+) of absence (−) of 0.5% v/v Triton X-100 for the indicated times and fluorescence measured with a fluorimeter. (D) Purification of SNAP-ABCG2 by metal affinity chromatography, following SMALP solubilisation. Fractions indicate whole cell membranes (M), SMALP-insoluble (I), SMALP-soluble (S), flow through (FT), wash (W) and elution (E). (E) Purification fractions containing purified SNAP-ABCG2 were concentrated by centrifugation. A preparation of material from cells not expressing ABCG2 was treated similarly. 10 μL of each of these samples was run on an 8% (w/v) polyacrylamide gel and stained InstantBlue. ABCG2 (identified *) and some contaminants were revealed.

Having confirmed that SMALP extraction preserved the dimeric form of ABCG2 as detected in live cells, we then determined whether FCS could be applied to quantify ABCG2:substrate interactions. Using the GFP-ABCG2 construct would limit this approach to fluorescent drugs whose spectra do not overlap with GFP. Construction of an ABCG2 fusion with a SNAP-tag protein, for which a range of different SNAP-ligands is available, would allow discrimination between ABCG2 and a wider range of fluorescent substrates. An ABCG2 variant with a His_6_-SNAP-tag at the N-terminus was expressed stably in mammalian HEK293T cells (Fig. 4A, left panel). Labelling of cells expressing His_6_-SNAP-ABCG2 with cell-permeable SNAP-Cell Oregon Green confirmed a predominant membrane localisation of the protein; specific labelling for the tagged ABCG2 variant was completely blocked by pre-incubation with non-fluorescent SNAP-Cell Block reagent (benzyl guanine; Fig. 4A middle and right panels). The function of this ABCG2 construct was validated with a drug transport assay [33], in which the cellular export of the fluorescent drug substrate mitoxantrone [47] was determined in the presence or absence of the inhibitor Ko143 [48]. Although Ko143 has some inhibitory activity towards ABCB1 we employed it at 1 μM, a concentration at which it is effectively ABCG2-specific [49]. Ko143 inhibited ~80% of mitoxantrone transport in His_6_-SNAP-ABCG2 expressing cells, with no significant transport in untransfected cells (Fig. 4B). This function was comparable to other N-terminally labelled ABCG2 constructs which have previously been studied [34,[50], [51], [52]] indicating that the SNAP tag does not impact negatively on either membrane trafficking or transport function of ABCG2.

His_6_-SNAP-ABCG2 was also labelled by SNAP fluorophores in membrane preparations (Fig. 4C) and this was not disrupted by the addition of membrane solubilising detergents (Triton X-100). Following labelling of membranes, we employed SMA to solubilise His_6_-SNAP-ABCG2 under identical conditions to those described earlier, and took advantage of the N-terminal hexahistidine tag to purify the protein using Co^2+^ affinity chromatography. SMALP-ABCG2 was concentrated to approximately 2 μM (Fig. 4D, E). Interestingly, and consistent with other reports (see refs within [28]), we observed that the affinity of SMA-encapsulated ABCG2 for metal chelated resins was lower than when the protein was solubilised in non-denaturing detergents. Presumably, some interaction between SMA and the His-tag results in either an occlusion of the tag or weaker binding to the resin. We observed that washing the resin in the absence of imidazole removed most contaminating proteins; using 20–50 mM imidazole washes was then sufficient to then elute the target protein from the resin. Gel analysis of purified protein showed a small number of contaminating species so we performed parallel purifications of untransfected HEK293T cells. This enabled both ABCG2-enriched and non-ABCG2 containing SMALPs to be prepared for subsequent FCS analysis (Fig. 4E).

### 3.4 Transport substrate binding to ABCG2 demonstrated by FCS

Currently, determination of drug binding to ABCG2 is restricted to radioligand binding of isotope labelled substrates. Though informative [20,21] these studies are restricted to the use of isotope labelled daunomycin which is only a substrate for the R482G/A/T mutant versions of ABCG2 [53]. Development of fluorescence based assays to study ABCG2 pharmacology would extend our ability to develop structure activity relationships for this multidrug pump. Purified, SMALP-encapsulated ABCG2 was used to determine the potential for pharmacological investigations of soluble ABCG2 in a membrane environment. In principle, the binding of a small, fluorescent substrate to ABCG2 should be accompanied by a significant reduction in its diffusion coefficient, as determined by the change in mass (~1 kDa to ~250 kDa; estimated mass of ABCG2 dimer in a SMALP-ed section of membrane, see e.g. [45]). As with previous FCS studies on purified proteins, this should yield a sufficient difference in the diffusion coefficient between fast-moving free ligand and slower-moving bound ligand to enable concentrations of free and bound drug to be determined from a single autocorrelation function [54].

**Fig. 5:**
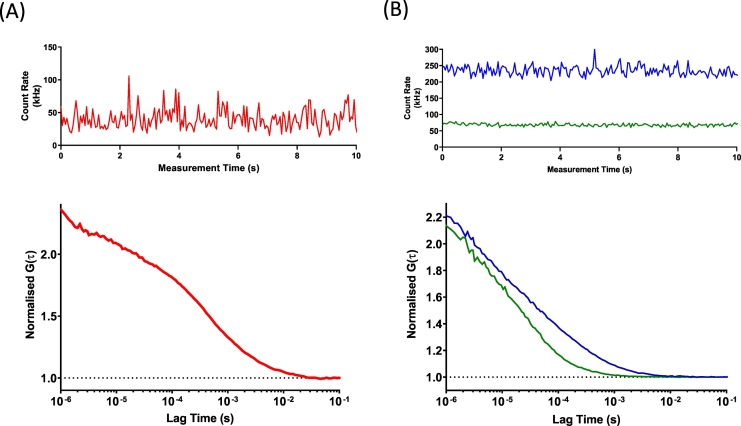
FCS analysis of SMALP-SNAP-ABCG2 and the binding of BODIPY-prazosin. (A) SMALPs were prepared from ABCG2-SNAP expressing cells, labelled with SNAP-Surface AlexaFluor 647 as described in Methods, and fluorescence fluctuations (*upper*) were recorded and autocorrelation curves generated. (B) Fluorescence fluctuations (upper) and subsequent autocorrelation curves (lower) were obtained for BODIPY-prazosin (50 nM) in the absence (green) and presence (blue) of unlabelled SMALP-ABCG2-SNAP, showing the presence of an additional slower component in the presence of purified ABCG2, representing bound BODIPY-prazosin. Data are from a single experiment representative of those described in Fig. 6.

**Fig. 6:**
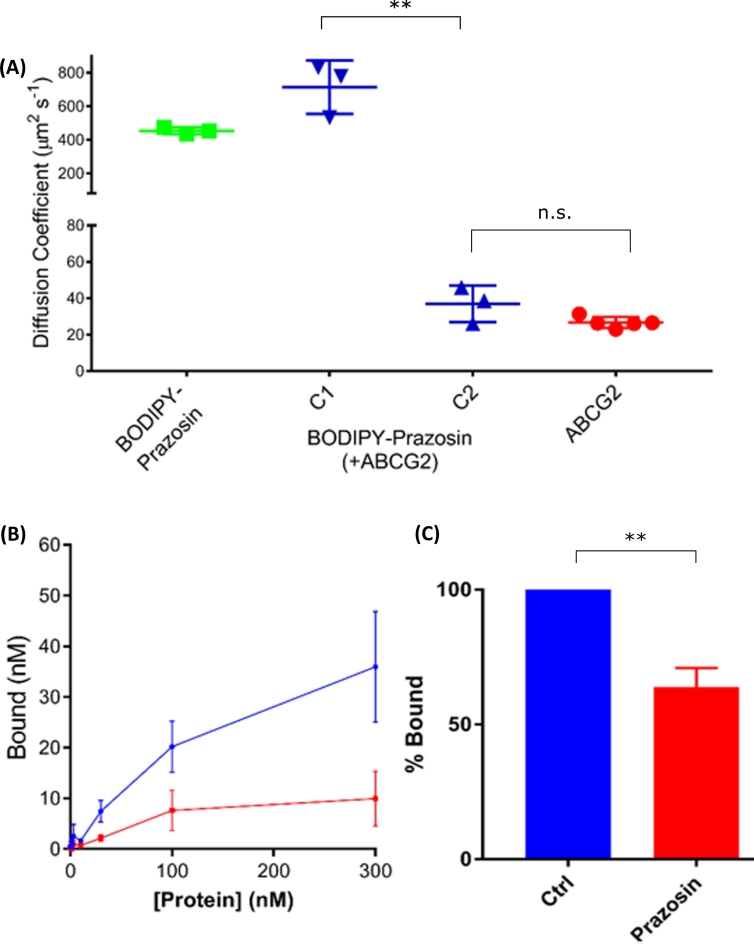
Quantification of BODIPY-prazosin free and bound components and determination of specific binding. (A) Autocorrelation analysis of BODIPY-prazosin in the presence of SMALP-ABCG2-SNAP yielded two components with differing diffusion coefficients: component C1 (‘free’) and C2 (‘bound’), respectively. Also shown are the diffusion coefficients for BODIPY-prazosin alone (green) and SNAP-Surface Alexa Fluor 647 labelled SMALP-ABCG2 (red) indicating these are comparable to the C1 and C2 respectively. Data shown are mean ± SD of 3 independent preparations, each measured in triplicate. Data were analysed by ANOVA and Tukey's multiple comparisons test and the significant difference between in diffusion co-efficient of bound BODIPY-prazosin (C2) compared to free BODIPY-prazosin (C2) is shown (**, p < 0.01). (B) BODIPY-prazosin (50 nM) was incubated with increasing concentrations of SMALP-ABCG2-SNAP (blue) or equivalent non-ABCG2-expressing SMALPs (negative control; red) for 30 min. Subsequent autocorrelation of fluorescence fluctuations allowed the concentrations of bound ligand (C2) to be determined in each sample. Data are shown as mean ± SEM, n = 3 independent preparations, each performed in triplicate. (C) SMALP-ABCG2-SNAP (100 nM) was incubated in the presence (prazosin) or absence (Ctrl) of 1 μM prazosin for 30 min prior to incubation with 50 nM BODIPY-prazosin for a further 30 min. Concentrations of bound BODIPY-prazosin were determined from autocorrelation analysis. Data were assessed for significance by unpaired *t*-test (** < 0.01) (mean ± SD, n = 3 independent experiments).

For these experiments, BODIPY-prazosin was used as a fluorescent transport substrate, as it has previously been shown to be a substrate for ABCG2 [50]. Initially, we characterised the diffusion of the two individual components in isolation. Fluorescence fluctuations were collected from solutions of AF647 labelled SNAP-ABCG2-SMALPs and analysed through autocorrelation analysis as previously described (Fig. 5A). Autocorrelation analysis revealed a single component with a diffusion coefficient of D = 26.7 ± 3.0 μm^2^s^−1^ (n = 5) which was similar to that seen for GFP-ABCG2 (Fig. 6A). Fluorescence fluctuations and subsequent autocorrelation analysis were also obtained for BODIPY-prazosin (500 nM) in assay buffer (green trace, Fig. 5B). This yielded a single component autocorrelation curve with a single diffusion component of 453.3 ± 11.9 μm^2^s^−1^ (n = 3), 17-fold faster than that of SNAP-ABCG2-SMALP particles (Fig. 6A).

ABCG2-SMALP (20–30 nM) was subsequently added to BODIPY-prazosin (500 nM) in assay buffer and incubated for 30 min. Fluorescence fluctuations were collected, and subsequent autocorrelation analysis revealed a biphasic curve. Restricting the fast component of this curve to the dwell time of free BODIPY-prazosin in solution (see Methods) revealed a second, slower diffusing component. This component had a diffusion coefficient of 37.0 ± 10.1 μm^2^s^−1^ (n = 3, P = n.s. compared to SNAP-ABCG2 alone) and represented BODIPY-prazosin bound to SMALP encapsulated ABCG2 (Figs. 5B; 6A).

Whilst these initial experiments clearly indicated binding of a proportion of the BODIPY-prazosin to SMALP particles encapsulating the SNAP-ABCG2, this binding could be specific (to ABCG2 itself) or non-specific (to lipid, SMA polymer for instance). Further experiments were performed to identify the specific component of the binding. In each case, absolute concentrations of free and bound ligand were determined following fitting of the autocorrelation curves to a two-component fit, with diffusion times defined based on those determined for free ligand and directly labelled SMALP.

Firstly, a comparison was made between the bound levels of ligands (component 2) in SMALPs containing SNAP-ABCG2 and equivalent amounts (in terms of protein content) of ‘empty’ SMALPs, i.e. those extracted from HEK293 cells not expressing ABCG2 (Fig. 4E). Over a range of protein concentrations, in the presence of 50 nM BODIPY-prazosin, the levels of bound ligand were significantly higher than those in empty SMALPs (Fig. 6B). This indicated that a significant proportion of the bound ligand detected in ABCG2 SMALPs represented specific binding to the ABCG2 protein.

Further confirmation that the bound component represented specific binding to ABCG2 was obtained by investigating the effect of unlabelled prazosin on levels of BODIPY-prazosin binding in ABCG2 containing SMALPs. As demonstrated in Fig. 6C, pre-incubation with 1 μM prazosin caused a significant decrease in the amount of bound ligand detected in FCS experiments. Determination of the specific binding and displacement of BODIPY-prazosin to wild type ABCG2 opens up the possibility of explore transport substrate and inhibitor interactions of this MDR pump.

Numerous studies have shown that there is a complex pharmacology for ABCG2 [21,55] that is beginning to be understood at the structural level through cryo-EM observations on the protein [[22], [23], [24]]. Determining how mutations (or naturally occurring single nucleotide polymorphisms) in ABCG2 contribute to altered substrate transport is easily achieved using flow cytometry [34,56,57] but direct quantification of how mutations/SNPs may affect the initial drug binding step can now be envisaged as possible using SMALPed ABCG2 and FCS. A similar FCS based approach has also been applied to another of the human multidrug pumps, ABCB1, and provides further evidence of the experimental possibilities of FCS studies of transporters in solution [58]. Importantly, given the observed evidence for ABCG2 to impact on drug pharmacokinetics [14] and the need to quantify how new drugs interact with ABCG2 [59], this technique – which employs small amounts of purified protein - may have the potential to address these quantitative aspects of drug:transporter interaction.

The following is the supplementary data related to this article.

Supplementary Fig. 1 Samples of SMA solubilised membranes were analysed by dynamic light scattering. Typical DLS traces shown from three independent repeats with 10 replicate reads per experiment. Supplementary Fig. 1

## Funding

AJH was funded by a BBSRC Doctoral Training Programme grant (BB/J014508/1) to the University of Nottingham.

## Author contributions

IDK, NDH, SJB conceived and designed the experiments. AJH carried out the experiments. DAB assisted with experimental work. AJH, IDK, NDH and SJB analysed the data and contributed to the final manuscript.

## CRediT authorship contribution statement

**Aaron J. Horsey:** Data curation, Investigation, Formal analysis, Visualization, Writing - original draft.**Deborah A. Briggs:** Investigation.**Nicholas D. Holliday:** Methodology, Conceptualization, Project administration, Supervision, Visualization, Writing - original draft, Writing - review & editing.**Stephen J. Briddon:** Methodology, Conceptualization, Project administration, Formal analysis, Supervision, Visualization, Writing - original draft, Writing - review & editing.**Ian D. Kerr:** Methodology, Conceptualization, Project administration, Supervision, Visualization, Writing - original draft, Writing - review & editing.

## Declaration of competing interest

The authors declare that they have no known competing financial interests or personal relationships that could have appeared to influence the work reported in this paper.
