# Supplementary material for: Application of fluorescence correlation spectroscopy to study substrate binding in styrene maleic acid lipid copolymer encapsulated ABCG2
Source: Biochim Biophys Acta Biomembr. 2020 Jun 1;1862(6):183218. doi: 10.1016/j.bbamem.2020.183218 (PMC7156912; doi:10.1016/j.bbamem.2020.183218)
Supplement: Supplementary Fig. 1 — Samples of SMA solubilised membranes were analysed by dynamic light scattering. Typical DLS traces shown from three independent repeats with 10 replicate reads per experiment. [file mmc1.pptx]

## Slide 1
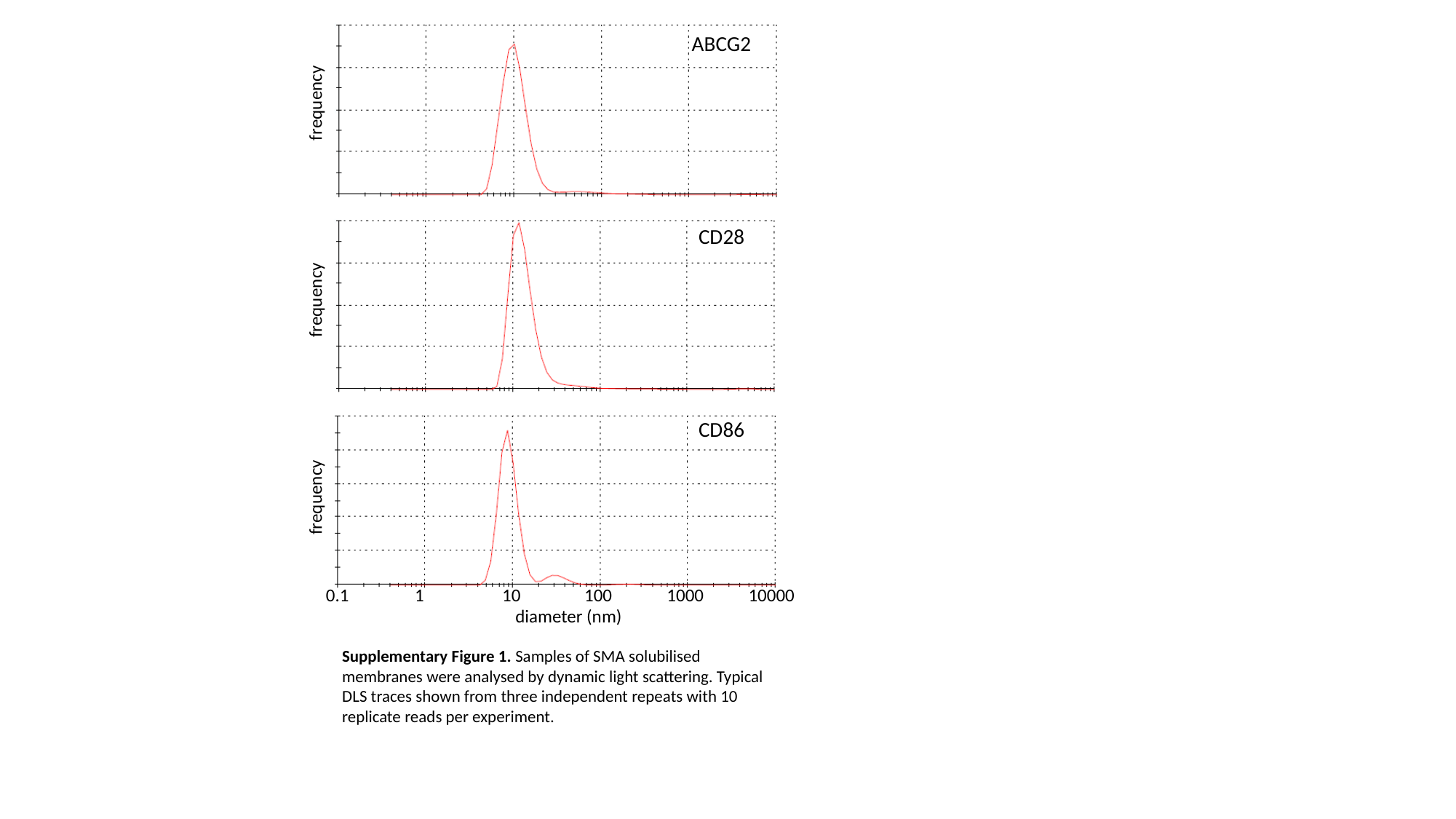

ABCG2
frequency
CD28
frequency
CD86
frequency
0.1
1
10
100
1000
10000
diameter (nm)
Supplementary Figure 1. Samples of SMA solubilised membranes were analysed by dynamic light scattering. Typical DLS traces shown from three independent repeats with 10 replicate reads per experiment.
